# Whole-body diffusion-weighted MRI: a new gold standard for assessing disease burden in patients with multiple myeloma?

**DOI:** 10.1038/leu.2015.338

**Published:** 2016-01-19

**Authors:** C Pawlyn, L Fowkes, S Otero, J R Jones, K D Boyd, F E Davies, G J Morgan, D J Collins, B Sharma, A Riddell, M F Kaiser, C Messiou

**Affiliations:** 1 The Institute of Cancer Research, London, UK; 2 Department of Haematology, The Royal Marsden Hospital NHS Foundation Trust, London, UK; 3 Department of Radiology, The Royal Marsden Hospital NHS Foundation Trust, London, UK; 4 Myeloma Institute, University of Arkansas for Medical Sciences, Little Rock, AR, USA; 5 CRUK Cancer Imaging Centre, The Institute Of Cancer Research, and The Royal Marsden Hospital NHS Foundation Trust, London, UK

Whole body magnetic resonance imaging (MRI) is now recommended for all patients suspected of having asymptomatic myeloma or solitary plasmacytoma, for the detection of disease defining lesions.^1, 2^ Whole body imaging has the potential to easily and painlessly assess the burden of disease throughout the whole marrow at different disease timepoints and may be predictive of disease outcome from diagnosis.^3, 4^ Conventional MRI has previously been shown to be more sensitive and specific than ^18^F-fluorodeoxyglucose positron emission tomography–computed tomography (FDG PET–CT) for the detection of small focal lesions and diffuse marrow infiltration.^5, 6^ Until recently, however, only FDG PET–CT offered a quantitative measurement in the form of standard uptake value. With the recent development of whole-body diffusion-weighted MRI (WB-DWI) for myeloma, a non-ionising radiation imaging modality for quantitative assessment of disease burden and therapy response has become available. WB-DWI uses the apparent diffusion coefficient (ADC) measurement to quantify disease.^7^ ADC is influenced by tissue microarchitecture but specifically relates to marrow cellularity.^8^ The speed, quantitative capabilities and superior sensitivity of WB-DWI compared with conventional MRI sequences^9^ have led to its adoption at several leading myeloma centres worldwide. Additional benefits include the assessment of skeletal complications and no intravenous contrast. Despite these advances there has been a lack of data directly comparing WB-DWI and FDG PET–CT to evaluate their relative merits.

Although quantifying tumour metabolism with FDG PET–CT is highly attractive, in reality standard uptake value measurements are profoundly influenced by numerous factors including serum glucose, uptake time, scanner calibration and reconstruction techniques. ADC measurements from diffusion-weighted MRI at different time points are not without its challenges and can be influenced by scanner manufacturer and field strength. However, ADC is felt to be less prone to equipment and physiological influences. Bone marrow ADC measurements have been achieved with a coefficient of variation of 2.8% in myeloma patients, suggesting excellent reproducibility^7^ and good inter-observer agreement (*K* 0.69) has also been reported.^10^

We have compared matched simultaneous assessments of myeloma using FDG PET–CT and WB-DWI and determined the relationship to bone marrow biopsy assessments of disease burden. This retrospective, single institution study was undertaken with approval of the institute review board. Patients who had undergone paired WB-DWI and FDG PET–CT scans for myeloma at any stage of disease were included. WB-DWI scans were obtained using an Avanto 1.5 T system (Siemens, Erlangen, Germany) and serial acquisition of contiguous body regions from skull vertex to knees using *b* values of 50 and 900/mm^2 ^s. FDG PET–CT studies were acquired using either a Gemini PET/CT (Philips, Best, The Netherlands) or a Biograph mCT S128 (Siemens). Patients are asked to fast for at least 4 h before the study. ^18^F-FDG (400 MBq) was injected i.v. and blood sugar levels were confirmed to be <10 mmol/l. Patients rested for 60 min prior to FDG PET–CT acquisition. Emission data and unenhanced CT were acquired from the skull vertex to the knees.

Scans were excluded if there was any evidence of a change in the clinical status of the patient between scans (that is, biochemical progression or commenced on treatment). Patients who had undergone paired scans more than once during the course of their disease were included provided their disease status had changed between scans. For each body region (skull, C spine, T spine, L spine, pelvis, ribs/other, long bones) in each patient scan, two radiologists (WB-DWI=CM, AR; FDG PET–CT=BS, LF) made a consensus assessment of disease burden using a previously described scoring system.^7, 11^ This scoring system is based on the presence of focal lesion number and size or diffuse disease, with a higher score indicating a greater burden of disease, and has previously been shown to have good inter-observer variability (Intraclass correlation coefficient 0.74).^11^

Whole body and per-region scores were compared. As the data were not normally distributed the Wilcoxon matched-pairs signed rank test was used to evaluate whether the scores for WB-DWI were significantly different from those for FDG PET–CT by body region and by patient. The value of *P*<0.05 was used as the cutoff for statistical significance in all tests. The Spearman's correlation coefficient was calculated to assess the correlation between scores. Patient clinical data were reviewed and bone marrow trephine results recorded to assess patients' laboratory estimates of disease burden. Bone marrow trephine results were compared with the assessment of disease infiltration at the iliac crests recorded by the radiologists. The percentage plasma cell infiltration (PC%) was correlated to the imaging score (Spearman's correlation) and compared with qualitative descriptions.

Twenty pairs of scans performed in 17 patients (11 male, 6 female; ages 42–72) were included. The scans took place within a median of 9.5 days (range 0–78), mean 14 days±18.15. There was a positive correlation between WB-DWI and FDG PET–CT scores (Spearman's *r*=0.35, 95% confidence interval (−0.12–0.69), *P*=0.13). However, the mean whole-body score for WB-DWI was significantly higher than for FDG PET–CT (WB-DWI Mean 17.65±12.24 vs FDG PET–CT Mean 8.45±8.70. *P*=0.0023), indicating higher sensitivity of WB-DWI than PET–CT for disease detection. In all regions, scores for WB-DWI (Table 1) were higher than FDG PET–CT with statistical significance in all except for pelvis and long bones.

Diffuse disease was reported in 37% (51/138) of regions imaged on WB-DWI scans compared with only 7% (10/140) on FDG PET–CT (2 regions in 1 patient were not included in the DWI scan). The regions reported as having diffuse disease on WB-DWI were reported on FDG PET–CT as ‘no disease' for most (36/51) regions (Figures 1a and b). Overall 3 patients were reported as being disease free on FDG PET–CT while having extensive (⩾3/7 regions) diffuse disease evident on WB-DWI, that is, representing a false-negative misdiagnosis by FDG PET–CT. These data demonstrate the clinically relevant superior sensitivity of WB-DWI in detecting the presence of diffuse disease over FDG PET–CT. To compare the ability of both modalities to detect focal lesions alone, whole-body scores were reanalyzed excluding diffuse disease. Mean scores for WB-DWI remained higher than for FDG PET–CT but this was not statistically significant, indicating at least equal sensitivity of WB-DWI for detection of focal lesions. (WB-DWI mean 7.45±10.32, FDG PET–CT mean 6.45±7.06, *P*=0.92).

For 10 WB-DWI scans demonstrating active disease a follow-up WB-DWI was available and showed a change in disease burden in response to therapy. This provides evidence of the potential utility of WB-DWI as a response assessment tool. Extramedullary disease not arising from bone was reported on one WB-DWI, in the liver, and was not identified on the corresponding FDG PET–CT scan. Resolution of this lesion on subsequent WB-DWI imaging, following treatment, confirmed this as true extramedullary myeloma. The liver, along with cranium, are areas where it is difficult for FDG PET–CT to pick up small lesions due to lower spatial resolution and relatively high background FDG activity, respectively.

About 15/20 pairs had bone marrow trephines with adequate tissue for the calculation of PC% performed close to the FDG PET–CT and WB-DWI scans. The PC% was better correlated to WB-DWI scores (Spearman's *r*=0.36 95% confidence interval (CI) (−0.20, 0.74) *P*=0.19) than FDG PET–CT (Spearman's *r*=0.20 95% CI (−0.36, 0.67) *P*=0.46). In 93% of patients, the trephine PC% was similar to the qualitative WB-DWI report of activity at the iliac crests compared with only 67% for FDG PET–CT. Furthermore if the trephine preceded imaging, the tract was more readily visible on WB-DWI scans compared with FDG PET–CT scans (Figure 1c).

We demonstrate that, in paired scans, WB-DWI detects a statistically significant higher burden of myeloma disease than FDG PET–CT. In the setting of the new International Myeloma Working Group consensus [1] detection of focal lesions on WB-DWI will lead to greater numbers of patients meeting criteria for active disease requiring treatment. The increased sensitivity and potential for quantification of diffuse marrow infiltration may provide further insight into this disease pattern in future studies. WB-DWI also outperforms FDG PET–CT in the assessment of disease infiltration of the iliac bones, essential for good clinical correlation of trephine results. All WB-DWI scan reports in our institution include an assessment of the burden of disease at the iliac crests, and relative to visible trephine tracts, to correct for the potential sampling error inherent in the use of single site bone marrow sampling and inform clinical management decisions.

In addition to this study we have recently investigated patient experience of WB-DWI. Twenty-eight patients were asked to report their experience using standardised questionnaires. With mean total scan length of 49 min, 24/28 (86%) patients found the overall experience not at all (11/24) or not too (13/24) unpleasant. About 27/28 (96%) patients reported no or little increase in their level of pain or discomfort and 26/28 (93%) would be happy to have the examination again, suggesting this imaging technique is acceptable to patients.

Our results demonstrate that WB-DWI is a highly sensitive quantitative imaging technique for detecting diffuse and multifocal marrow infiltration in patients with myeloma and can be used to assess for trephine sampling error. This supports current International Myeloma Working Group guidance^1^ that MRI should be the imaging investigation of choice for detection of myeloma marrow infiltration given its higher sensitivity than FDG-PET–CT. Further studies are needed to determine the clinical significance of WB-DWI-positive/FDG-PET-negative lesions.

## Figures and Tables

**Figure 1:**
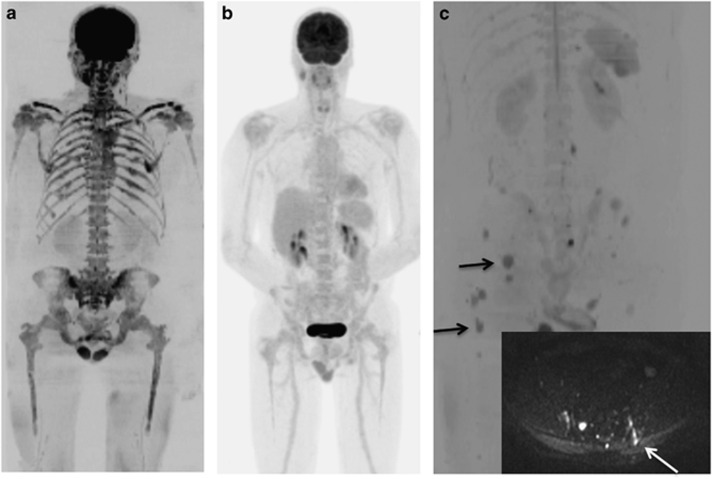
WB-DWI improves detection of diffuse marrow infiltration compared with FDG PET–CT and detects trephine sampling error. WB-DWI b900 maximum intensity projection image (**a**) and FDG PET–CT (**b**) in the same patient. WB-DWI demonstrates diffuse infiltration of the bone marrow; FDG PET–CT shows no evidence of disease. WB-DWI b900 maximum intensity projection image (**c**) demonstrates multifocal disease (examples shown by arrows). Axial b900 image (superimposed) shows the trephine tract (arrow), which does not sample a focal lesion.

**Table 1:** Observer scores for whole-body diffusion-weighted imaging (WB-DWI) are higher than for FDG PET–CT for the whole body and in all body regions

	*Score mean (*±*s.d.)*		P-*value*^a^
	*FDG PET–CT*	*WB-DWI*	
Whole body	8.45 (±8.70)	17.65 (±12.24)	0.002

*Region*			
C spine	0.47 (±1.17)	1.68 (±1.92)	0.016
T spine	1.20 (±1.64)	2.60 (±2.04)	0.011
L spine	1.00 (±1.62)	2.50 (±2.04)	0.007
Pelvis	2.40 (±2.54)	3.30 (±1.95)	NS, 0.13
Long bones	1.85 (±2.35)	2.80 (±2.71)	NS, 0.19
Skull	0.21 (±0.63)	1.95 (±1.96)	0.004
Ribs/other	1.35 (±1.90)	3.00 (±1.97)	0.006

Abbreviations: FDG PET–CT, ^18^F-fluorodeoxyglucose positron emission tomography–computed tomography; NS, not significant.

a Wilcoxon matched-pairs signed rank test.
